# Fœtid Sweating of the Feet

**Published:** 1882-02

**Authors:** 


### Fœtid Sweating of the Feet.

Willcox, referring to the above article, says that he does not believe that the bad odor is occasioned by any putrefactive changes in the sweat, and says that the same odor does not occur in sweating of the axilla, scrotum, or perinæum. He claims to have been successful in treating bromidrosis of the feet by means of the application of strips of emplastrum saponis, or emplastrum plumbi. The plaster should be changed every three or four days, and, in bad cases, two layers applied. At the end of a week he has found the skin of a natural color and dry, the odor ceasing from the first.

Thin, in a subsequent article, while confirming much of what Willcox says, finds that the local application of adhesive plaster has failed in his hands. He says that the offensive odor is occasioned by the mixture of serum with the sweat, and also that the same smell would be found in sweating of the perinæum, or scrotum, if the parts were chafed.

Clement Hawkins orders a foot-bath every night, to which an ounce of alum has been added. Afterward a lotion is to be applied to the red patches, made as follows: ℞ Plumbi oxidi rubr. gr xv, liquor plumbi subacetatis (French Pharm.) ℥i; pound the red oxide of lead in a mortar until finely divided, and then gradually add the subacetate. H. says that a cure will be effected in from two to three weeks.—*British Med. Journal.*

